# Some assembly required: Redefining the mitotic checkpoint

**DOI:** 10.1080/23723556.2017.1314238

**Published:** 2017-04-12

**Authors:** John C. Meadows, Jonathan B. A. Millar

**Affiliations:** a Division of Biomedical Sciences, Warwick Medical School, University of Warwick, Coventry, UK; b Institute of Advanced Study, University of Warwick, Coventry, UK

**Keywords:** Anaphase, chromosome passenger complex, microtubule, mitosis, spindle assembly checkpoint

## Abstract

The spindle assembly checkpoint (also known as the spindle or mitotic checkpoint) is a surveillance system that ensures fidelity of chromosome segregation. Here we suggest, in light of historical and more recent evidence, that this signaling system monitors kinetochore attachment and spindle assembly by two distinct, but functionally overlapping, pathways.

## Abbreviations

APC/C: Anaphase promoting complex/cyclosome
BBC: BubR1-Bub3-Cdc20
CPC: Chromosome passenger complex
KEN: Lysine-glutamic acid-asparagine
MCC: Mitotic checkpoint complex
PP1: Protein phosphatase 1
SAC: Spindle assembly checkpoint
MAD: Mitotic arrest deficient
BUB: BUdding uninhibited
MPS: Monopolar spindle
KNL: Kinetochore NulL
KLP: Kinesin-like protein
SGO: ShuGOshin

## 

Aneuploidy, or an incorrect number of chromosomes, is a central hallmark of cancer cells and a driver of cancer evolution.^1^ Eukaryotes have evolved a surveillance mechanism, termed the spindle assembly checkpoint (SAC; also referred to as the spindle checkpoint or mitotic checkpoint), to arrest the cell cycle in metaphase until all chromosomes are attached to microtubules that emanate from opposite spindle poles, a process referred to as chromosome bi-orientation. Classical laser ablation demonstrates that even a single unattached kinetochore is sufficient to prevent progression into anaphase.^2^ Components of the SAC were first discovered over 25 years ago in genetic screens for mutants that failed to arrest in mitosis in budding yeast.^3^ These include the Mad1, Mad2, Mad3(BubR1) and Bub3 proteins and the Bub1 and Mph1(Mps1) kinases. It is now known that SAC proteins are not only strongly conserved throughout evolution, but are mutated or display altered expression in a variety of human cancers,^4^ and that deregulated SAC activity drives tumor progression.

Our understanding of how the spindle checkpoint delays anaphase onset has improved enormously in recent years. Interaction of free Mad2 (O-Mad2) with kinetochore-bound Mad1-Mad2 complex catalyzes a conformational change in O-Mad2 to C-Mad2, which promotes interaction with Mad3(BubR1)-Bub3-Cdc20 to form the mitotic checkpoint complex (MCC), a potent inhibitor of the anaphase promoting complex/cyclosome (APC/C).^3^ Once chromosomes are correctly bi-oriented, the SAC is silenced and MCC is disassembled. This permits de-repression of APC/C, an E3 ubiquitin ligase, which catalyzes the ubiquitination of Securin and Cyclin B and their subsequent destruction by the proteasome. This triggers activation of Separase and the cleavage of Cohesin and inactivation of Cyclin B-Cdk1 kinase, which in turn trigger loss of sister chromatid cohesion and progression into anaphase, respectively.^3^ By contrast, it is much less clear as to what the SAC actually senses. Indeed, a long debate has been had as to whether the SAC is activated when kinetochores are not attached to spindle microtubules or when they are not under tension or both. Recent evidence that the Mad1-Mad2 complex binds to two distinct receptors on human kinetochores, the KNL1-Bub3-Bub1 (KBB) complex and the Rod-Zwilch-Zw10 (RZZ) complex,^5^ has re-ignited this debate. At present, it is unclear what mechanical event each of these receptors detects. Regardless, it is generally accepted that the SAC monitors some aspect of kinetochore microtubule interaction, but does not monitor spindle assembly *per se*. As such, the original name is a misnomer and requires redefinition.

The establishment of chromosome bi-orientation during early mitosis requires the activity of the Aurora B kinase, a component of the chromosome passenger complex (CPC), which binds the inner centromere region of chromosomes. When kinetochores are not under tension Aurora B kinase destabilizes inappropriate (e.g. merotelic or syntelic) microtubule-kinetochore attachments by phosphorylating components of the outer kinetochore. When tension is applied across the sister chromatids (centromere stretch), the outer kinetochore is pulled away from the inner centromere, thereby separating Aurora B kinase from its substrates. This promotes dephosphorylation of outer kinetochore proteins by type-1-phosphatase (PP1) and stabilization of tension-bearing microtubule-kinetochore attachments. In many species, including fission yeast, Aurora B kinase is also directly required to maintain the SAC signal independent of microtubules and the process of chromosome bi-orientation. SAC silencing requires association of PP1 to the N-terminus of the KNL1 kinetochore protein and Kinesin-8 motors.^6^ In this manner, the processes of chromosome bi-orientation and checkpoint silencing at the kinetochore are coupled, although the mechanisms involved are not fully defined.

In mammalian cells, re-localization of the CPC to the spindle mid-zone during anaphase requires MKLP2^7,8^ a member of the kinesin-6 family. Consistently, we found that Klp9, kinesin-6 in fission yeast, binds CPC during anaphase and is required for accurate CPC re-localization to the spindle midzone, and that both processes are negatively regulated *via* Cdk1 phosphorylation.^9^ Surprisingly, however, fission yeast cells lacking Klp9 display a protracted delay over anaphase onset despite having no detectable defect in chromosome bi-orientation. We traced this effect to an inability of Klp9 to bind CPC, rather than due to its motor activity, which is required for spindle elongation. More surprisingly, the delay in anaphase onset in *Δklp9* cells is dependent on some (Mad3, Bub1, and Mph1) but not all (Mad1, Mad2, Bub3) components of the SAC.^9^ This is in stark contrast to the canonical kinetochore attachment checkpoint, which strictly requires recruitment of Mad1 and Mad2 to unattached kinetochores. Secondly, while both the KEN boxes in Mad3 are required for the checkpoint response to unattached kinetochores, we find that only the first, but not second, KEN box of Mad3 is required for delaying anaphase onset in the absence of Klp9.^9^ Moreover, we were able to detect a Mad3-Cdc20 complex in the absence of Mad2, indicating that MCC is not the only functional inhibitor APC/C *in vivo*. This is intriguing as an analogous complex, which has been detected in human cells, that contains BubR1-Bub3-Cdc20 (BBC), but not Mad2, is a weak inhibitor of APC/C *in vitro*. Together these results suggest that there are two distinct mechanisms to inhibit the APC/C in fission yeast, one of which is dependent on unattached kinetochores and a second, which is revealed when re-localization of CPC to Klp9 (kinesin-6) is disrupted.

So, what might this second pathway monitor? Notably, pre-anaphase spindles frequently collapse in the absence of Klp9, indicative of a problem in spindle assembly. Similarly, spindles frequently collapse in cells lacking Ase1/PRC1, an anti-parallel microtubule bundling protein, and anaphase onset is delayed in Δ*ase1* cells by a mechanism that also requires Mad3, Bub1, and Mph1 but not the Mad1, Mad2, or Bub3 checkpoint proteins.^10^ Is it possible that this alternative Mad2-independent pathway actually monitors spindle assembly and, if so, how? Notably, we find that Sgo2 (Shugoshin 2), which is partly required for association of CPC to inner centromeres, is required for the delay in anaphase onset in the absence of Klp9, but is completely dispensable for the canonical kinetochore attachment checkpoint. Since the inner centromere region occupies the space between two opposing kinetochores, it would be juxtaposed to the lateral face of the anti-parallel pole-to-pole spindle microtubules when under tension by kinetochore-associated microtubules. This would occur when all sister chromatids are amphitelically attached and when microtubule forces applied to opposing kinetochores are balanced at the metaphase plate. One possibility is that interaction of centromere associated Sgo2-CPC (Aurora B) kinase with Klp9 terminates phosphorylation of the Mad3-Cdc20 complex when all chromosomes congress to the spindle equator. The Mph1 kinase may simply be required to phosphorylate the MELT motifs of KNL1 to target Bub1 kinase to kinetochores, which is required to phosphorylate histone H2A to load Sgo2-CPC to centromeres. Clearly, further work will be required to test this and other possibilities, and the extent to which this alternative APC/C inhibitory pathway is conserved in higher eukaryotes. What is certain is that, 25 years on from its initial discovery, the functional complexity of the SAC necessitates a careful re-evaluation of its name to help us dissect it further.
